# Reduced tissue inhibitor of metalloproteinase-2 expression is associated with advanced medullary thyroid carcinoma

**DOI:** 10.3892/ol.2013.1767

**Published:** 2013-12-19

**Authors:** SIMONE MAGAGNIN WAJNER, CLARISSA CAPP, BEATRIZ ASSIS BRASIL, LUISE MEURER, ANA LUIZA MAIA

**Affiliations:** 1 Thyroid Section, Endocrine Division, Hospital de Clínicas de Porto Alegre, Universidade Federal do Rio Grande do Sul, Porto Alegre, RS 90035-003, Brazil; 2 Department of Pathology, Hospital de Clínicas de Porto Alegre, Universidade Federal do Rio Grande do Sul, Porto Alegre, RS 90035-003, Brazil; 3 Experimental Research Center, Hospital de Clínicas de Porto Alegre, Universidade Federal do Rio Grande do Sul, Porto Alegre, RS 90035-003, Brazil

**Keywords:** matrix metalloproteinases, tissue inhibitor of metalloproteinase, medullary thyroid carcinoma

## Abstract

Matrix metalloproteinases (MMPs) are enzymes for extracellular matrix remodeling that are involved in tumor growth, progression and metastasis. Among them, MMP-9 has been implicated in tumor angiogenesis. Tissue inhibitor of matrix metalloproteinase (TIMP)-2, a member of the family of MMP inhibitors, induces apoptosis and inhibits various stages of angiogenesis. Previous studies analyzing the expression of MMP-9 and TIMP-2 in medullary thyroid carcinoma (MTC) are scarce. The aims of the current study were to evaluate MMP-9 and TIMP-2 expression in MTC samples and correlate the results with clinical parameters. Paraffin-embedded samples from 77 MTC patients were evaluated for expression by immunohistochemistry. The clinical data in medical records were retrospectively reviewed. In total, 77 patients aged 35.6±17.1 years were enrolled. Of these patients, 36 had hereditary disease (46.8%). Immunohistochemical staining for MMP-9 and TIMP-2 was detected in 89.6 and 93.5% of the samples, respectively. The expression of MMP-9 was not found to correlate with clinical parameters, although, a trend toward a correlation between MMP-9 and distant metastasis was observed (P=0.053). By contrast, TIMP-2 staining was found to correlate with age at diagnosis (P=0.026) and negatively correlate with tumor size and tumoral stage (P=0.002 and P=0.001, respectively). Notably, the highest levels of TIMP-2 expression were observed in patients with intrathyroidal disease. The MMP-9 enzyme involved in extracellular matrix remodeling is overexpressed in MTC lesions and may contribute to tumor vascularization and growth. Reduced levels of TIMP-2 expression may be implicated in tumor progression and spread of disease.

## Introduction

Human matrix metalloproteinases (MMPs) are endopeptidases involved in the regulation of cell growth, migration and remodeling of the extracellular matrix. MMPs degrade matrix and non-matrix components and are regulated by several physiological inhibitors and upregulators. MMP deregulation facilitates the invasion of tumor cells into the surrounding connective tissue and vessels (1). Previous studies performed on several human neoplastic tissues have demonstrated a correlation between increased MMP expression and tumor invasion, metastatic spread, tumor recurrence and low survival rate (2). These proteolytic enzymes share a similar structure and are classified based on their substrate specificity. Accordingly, MMPs have been divided into collagenases, gelatinases, stromelysins and matrilysins. MMP-9, a member of the gelatinase group, not only readily digests denatured collagens and gelatins, but also plays a particular role in angiogenesis since it increases the bioavailability of proangiogenic factors (3–5).

The tissue inhibitors of metalloproteinases (TIMPs) constitute a family of four members that regulate MMPs through endogenous protease inhibition and cell surface activation regulation (2,6,7). In addition to this regulatory activity, TIMPs have multiple effects on cell growth, apoptosis and differentiation (6) through an MMP-independent mechanism (8). TIMP-2 induces apoptosis and inhibits various stages of angiogenesis (9,10). During cancer progression, high levels of TIMP-2 are associated with the inhibition of tumor growth, angiogenesis, invasion and metastasis, secondary to the inhibition of endothelial cell migration (11,12). In a previous cell model, TIMP-2 overexpression was shown to be cytostatic and prevent local invasion (13). As tumors progress, TIMP-2 expression levels are decreased or absent in several types of human cancer, particularly in invasive and metastatic tumors (12).

Medullary thyroid carcinoma (MTC) arises from parafollicular or C cells and accounts for 3–4% of all types of thyroid cancer. MTC may occur sporadically (75%) or through a hereditary mechanism caused by gain-of-function germline mutations in the RET proto-oncogene. RET molecular analysis is now considered essential in MTC management, since early diagnosis improves prognosis and allows adequate genetic counseling (14–16). The 10-year disease-specific survival rate of patients with MTC is ~75% (17). Currently, the only curative approach for MTC is surgical resection of the tumor, as it shows limited response to radiotherapy and/or chemotherapy. MTC tends to metastasize early via angioinvasion and hematogenous spread (16–19).

MMP and TIMP members have been shown to be upregulated in differentiated thyroid carcinoma (7,20), but little is known concerning their role in the pathogenesis or clinical presentation of MTC. The search for alternative treatments for metastatic disease has been intensified in the last decade based on new knowledge of the molecular biology of these tumors. Thus, characterizing these molecules may be useful in the development of new therapeutic strategies. The present study evaluated the expression of MMP-9 and TIMP-2 in MTC, and examined the correlation between the clinical features and the expression levels of these angiogenic factors.

## Materials and methods

### Thyroid tissue

The samples comprised of 77 specimens with histopathological/immunohistochemical diagnosis of MTC, which were obtained from patients attending the Endocrine or Head and Neck Divisions at the Hospital de Clínicas de Porto Alegre (university-based hospital; Porto Algre, Brazil) between 1997 and 2011. RET germline mutations were identified by standard procedure, as previously described (21). Sporadic MTC was diagnosed based on the absence of family history and known germline RET point mutations in exons 8, 10, 11 or 13–16. The clinical data in medical records were retrospectively reviewed. The Ethics Committee at the Hospital approved the study protocol (no. 10-0068).

For patients with clinical or biochemical evidence of MTC, the surgical procedure consisted of total thyroidectomy with varying cervical neck dissection procedures. For asymptomatic gene carriers with no abnormalities on cervical ultrasonography examination and normal serum calcitonin levels, prophylactic thyroidectomy was recommended. Tumor staging was performed according to the International Union Against Cancer tumor-node-metastasis (TNM) classification (22). Patients with suspicious distant metastasis (i.e., the presence of local metastases and/or serum calcitonin levels >150 pg/ml) underwent imaging examination (cervical, thoracic and abdomen CT or liver magnetic resonance imaging, as well as bone scintigraphy). Individuals with undetectable calcitonin and carcinoembryonic antigen (CEA) levels and normal physical examinations were considered to be in complete biochemical remission and were monitored annually without additional imaging, unless a change in exam results, symptoms or laboratory values was noted (16).

### Somatic M918T mutation analysis

For sporadic MTC patients, the frequency of somatic M918T mutation was analyzed. The MTC samples were material paraffin-embedded formalin-fixed tissue blocks. DNA was extracted using the Magnesil Genomic Fixed Tissue system (Promega Corporation, Madison, WI, USA) according to the manufacturer’s instructions. Exon 16 was amplified by polymerase chain reaction using 100–300 ng/ml DNA in a reaction mix (25 ml) containing 20 mM Tris-HCl (pH 8.0), 50 mM KCl, 2 mM MgCl_2_, 0.2 mM dNTPs, 0.2 mM each primer and 1.25 U Platinum Taq DNA Polymerase (Invitrogen Life Technologies, Carlsbad, CA, USA). The running profile of the amplifications and restriction fragment length polymorphism analysis were similar to those described previously for genomic DNA (21).

### Immunohistochemistry (IHC) analysis

IHC was performed on thin sections (3 μm) of previously formalin-fixed and paraffin-embedded tissues. The antibodies used were polyclonal rabbit antihuman vascular endothelial growth factor (VEGF)-A (clone VG1; M7273; Dako, Carpinteria, CA, USA) and monoclonal mouse anti-human VEGF receptor (VEGFR)-2 (A-3; SC-6251), TIMP-2 (YY6; sc80366) and MMP-9 (2C3; sc-21733) antibodies (Santa Cruz Biotechnology Inc., Santa Cruz, CA, USA). MTC samples were submitted to a routine immunohistochemical technique, which included deparaffinization and rehydration, antigenic recovery, inactivation of endogenous peroxidase and blockage of non-specific reactions. Primary antibodies were incubated overnight at 4°C at dilutions of 1:400 (VEGF-A), 1:200 (VEGFR-2) and 1:100 (TIMP-2 and MMP-9), followed by the application of streptavidin-horseradish peroxidase conjugate (LSAB; Dako) and diaminobenzidine tetrahydrochloride (DAB kit; Dako). The positive controls were human tissues, including skeletal muscle for VEGF-A, intestinal tumor for VEGFR-2, lung for TIMP-2 and heart for MMP-9. The negative control was obtained by omission of the primary antibody.

The intensity of VEGF-A, VEGFR-2, MMP-9 and TIMP-2 staining in each lesion was determined and quantified according to the following grades: 0, absent (−); 1, weak (+); 2, moderate (++); and 3, strong (+++). Grading was based on the predominant staining characteristics of the tumor. The slides were examined using an Olympus BX51 microscope with an Olympus QColor 5 camera (Olympus America Inc., Melville, NY, USA). The slides were independently read by two blinded and experienced pathologists, who were unaware of the respective clinicopathological data. When the two experts differed in their interpretations, they consulted with each other to reach a consensus.

### Statistical analysis

The data are presented as the median and interquartile range (IQR). Baseline characteristics were compared using the χ^2^ test for qualitative variables and the Mann-Whitney U test for quantitative variables. Spearman’s coefficient or the Mann-Whitney U tests were used to assess the correlation between angiogenic marker expression (VEGF-A, VEGFR-2, TIMP-2 or MMP-9) and age at surgery, tumor size, TNM stage and disease outcome. P<0.05 was considered to indicate a statistically significant difference. The Statistical Package for the Social Sciences 18.0 professional software (SPSS, Inc., Chicago, IL, USA) was used for statistical analysis.

## Results

### Patients

The clinical and oncological features of the 77 patients included in this study are shown in Table I. The median age at diagnosis was 35.6 years (IQR, 2.5–83.3 years) and 45 (58.4%) of the patients were female. In total, 36 (46.8%) patients had hereditary MTC, whereas 41 (53.2%) patients had the sporadic form of the disease. Of these patients, 34 had MEN 2A and two were found to have MEN 2B syndrome. The RET mutations identified in MEN 2A patients were as follows: C634Y (25 individuals; 73.5%), C634R (four individuals; 11.7%), C618R (three individuals; 8.8%) and E768D (two individuals; 5.8%). The two patients with MEN 2B presented with the characteristic phenotype and a mutation in codon 918.

At the time of surgery, 34 (46.6%) patients presented with lymph node disease and 14 (18.9%) exhibited distant metastases. The median calcitonin level was 262.0 pg/ml (IQR, 28.0–953.6 pg/ml) and the median CEA level was 14.6 ng/ml (IQR, 2.4–52.6 ng/ml). In total, 17 patients were diagnosed with tumor stage I (22.1%), 24 with stage II (31.2%), 22 with stage III (28.6%) and 14 with stage IV (18.2%). In addition, 39 (61.9%) patients were considered free of disease following a follow-up period of 6.96±4 years.

### MMP-9 and TIMP-2 expression in MTC

**Figure 1:**
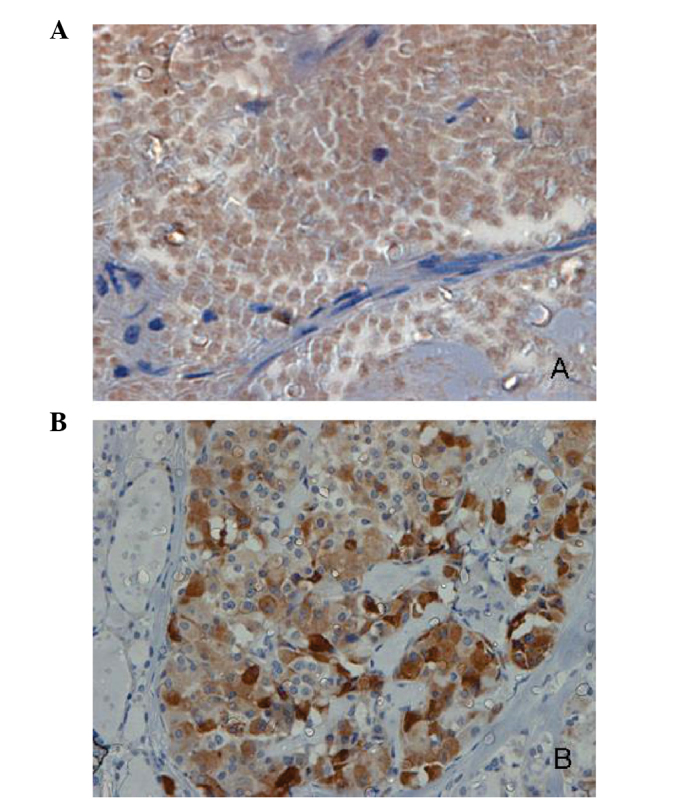
Immunohistochemical detection of (A) matrix metalloproteinase-9 and (B) tissue inhibitor of metalloproteinase 2 in the cytoplasm of the malignant cells of a medullary thyroid carcinoma sample, as shown by the brown staining (magnification, ×200).

Immunohistochemical staining for MMP-9 and TIMP-2 were detected in 69 (89.6%) and 72 (93.5%) out of the 77 samples analyzed, respectively (Fig. 1A and B). As predicted, positive MMP-9 and TIMP-2 immunoreactivity was detectable in the cytoplasm of thyroid cancer cells, but rarely in stromal cells or surrounding healthy thyroid tissue.

**Figure 2:**
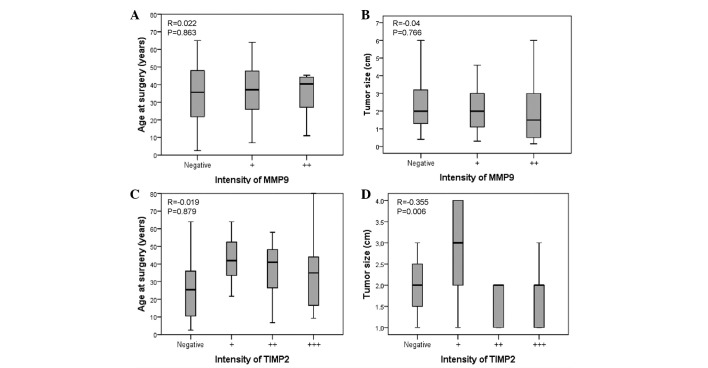
Correlations between MMP-9 staining and (A) age at surgery and (B) tumor size. Correlations between TIMP-2 staining and (C) age at surgery and (D) tumor size. MMP-9, matrix metalloproteinase; TIMP-2, tissue inhibitor of metalloproteinase 2.

The expression of MMP-9 was not found to correlate with age or tumor size (P=0.8 and P=0.76, respectively; Fig. 2) or TNM stage (P=0.37; Table II). However, a trend toward an association, although not statistical significant, was observed between MMP-9 and baseline levels of calcitonin (r=0.296; P=0.06) and distant metastasis (P=0.053) (Table III).

TIMP-2 intensity was not found to correlate with age (P=0.8; Fig. 2C), but was found to negatively correlate with baseline levels of calcitonin (r=−0.327; P=0.036), tumor size (r=−0.355; P=0.006; Fig. 2D) and tumoral stage (r=−0.395; P=0.001; Table II). The highest TIMP-2 expression was observed in samples from patients without local (r=−0.423; P=0.0001) or distant (r=−0.416; P=0.001) metastasis (Table III).

### VEGF and VEGFR-2 expression in MTC

**Figure 3:**
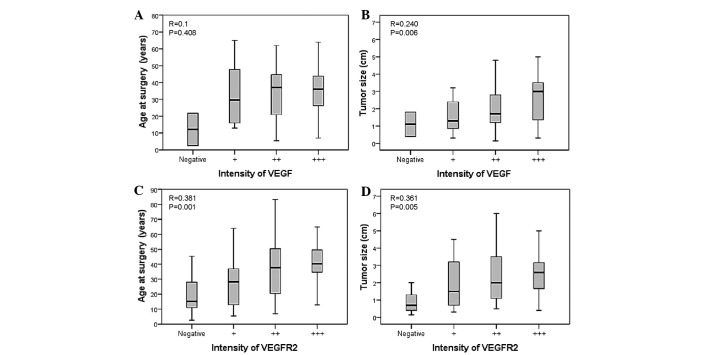
Correlations between VEGF staining and (A) age at surgery and (B) tumor size. Correlations between VEGFR-2 staining and (C) age at surgery and (D) tumor size. VEGF, vascular endothelial growth factor; VEGFR-2, vascular endothelial growth factor receptor 2.

The expression of VEGF and its receptor was also investigated. The analysis of the expression of these angiogenic molecules with clinical parameters did not demonstrate a correlation with age (P=0.4; Fig. 3A). Nevertheless, a positive correlation was identified between VEGF-A expression and tumor size (r= 0.240; P=0.006; Fig. 3B). In addition, VEGFR-2 was found to positively correlate with age at surgery (r=0.381; P=0.001; Fig. 3C) and tumor size (r=0.361; P=0.005; Fig. 3D). A positive correlation was also identified between VEGFR-2 and TNM stage (r=0.397; P=0.002). Notably, an inverse correlation was identified between TIMP-2 and VEGF-A expression (r=−0.270; P=0.024).

### Sporadic and hereditary MTC tumors

Since there are hereditary and sporadic forms of MTC, the two groups were analyzed separately. Patients with the hereditary form of the disease had a mean age of 26.2±16.7 years. In this group, 39.9% of the patients were diagnosed with stage I of the disease, 33.3% with stage II, 22.2% with stage III and 2.8% with stage IV. Compared with the hereditary group, patients with sporadic MTC were older (41.9±15 years old; P<0.0001) and presented with more advanced disease at diagnosis (stage I, 2.4%; stage II, 31.7%; stage III, 24.4%; and stage IV, 31.7%; P<0.0001). No significant difference was identified between the groups with regard to MMP-9 staining (P=0.654; Table IV). Nonetheless, higher TIMP-2 expression was observed in patients with hereditary disease (P=0.001; Table IV).

In the sporadic form of the disease, the analysis of clinical parameters demonstrated an inverse correlation between TIMP-2 staining and tumor size (r=−0.429; P=0.02) and tumor stage (r=−0.475; P=0.006) (Table IV). In addition, patients with the sporadic form and tumors restricted to the thyroid were found to present the highest levels of TIMP-2 (P=0.018).

The following step was to identify the somatic RET M918T mutation in the sporadic group, since it has been shown that the presence of the missense somatic RET mutation correlates with aggressive disease. In total, 31 DNA samples were available for analysis and 25 samples (80.6%) were found to exhibit somatic M918T. The presence of the somatic mutation was not found to correlate with the expression of MMP-9 (P=1.00) or TIMP-2 (P=0.88).

## Discussion

The current study examined the expression of pro-invasive factors in MTC. MMP-9 and TIMP-2 expression were observed in ~90% of the samples. While MMP-9 did not show any correlation with clinical parameters, TIMP-2 immunoreactivity was found to inversely correlate with tumor size and stage of the disease at diagnosis. Notably, the samples with more intense TIMP-2 staining were from patients without local or distant metastasis.

Cancer cells degrade basement membranes and invade tissues. TIMPs, secreted proteins that complex with individual MMPs, regulate the functional activity and activation of individual MMPs (23). MMP-9 is a functional component of the angiogenic switch during carcinogenesis as it increases the bioavailability of pro-angiogenic growth factors, including VEGF-A (24). Conversely, TIMP-2 inhibits not only VEGF-induced VEGFR-2 phosphorylation, but also endothelial cell growth in response to fibroblast growth factor-2 (FGF-2), likely through the inhibition of FGF-2-induced ERK1/2 signaling (2,25).

Previously, several studies have shown augmented MMP-9 expression in differentiated thyroid carcinoma, demonstrating a correlation between MMP-9 levels and lymph node metastasis (20,26). Nevertheless, other studies have failed to demonstrate increases in MMP-9 expression in papillary thyroid carcinoma (PTC) (27). Studies analyzing TIMP-2 have also shown controversial results. While the expression of this molecule has been found to positively correlate with tumor size, tumoral stage and vascular invasion in PTC (20), contradictory results have been observed in other carcinomas, in which TIMP-2 has been shown to be more frequently associated with localized tumors than regional or distant metastases (28,29).

Few studies have analyzed MMP-9 and TIMP-2 expression in MTC. A previous small study that evaluated 10 cases of MTC showed weak MMP and TIMP immunostaining (30). An additional study that evaluated 37 MTC samples found that TIMP-2 expression did not correlate with any clinical parameter at diagnosis (31). This is consistent with the results of the current study, which did not identify a correlation between MMP-9 expression and clinical parameters. However, the trend toward a correlation between MMP-9 with baseline calcitonin levels and distant metastasis suggests that the lack of correlation may be due to the large number of patients in the early stages of disease (I and II). Furthermore, the various histological origins between tumors may also be implicated in a putative and different action of MMP-9 in MTC.

As aforementioned, TIMPs have been implicated in promoting and inhibiting cell growth, suggesting that the specific effects of TIMPs on cell fate depend on the cell context and specific model system under study. Although TIMP-2 has been associated with large tumor size and high invasiveness in PTC (20), positive TIMP-2 staining is significantly higher in localized colorectal tumors and negative in the invasive forms (29). This also appears to be the case in MTC, in which an inverse correlation was observed in the current study between TIMP-2 staining and baseline calcitonin, tumor size, tumor stage and distant metastases, suggesting that increased levels of TIMP-2 may be a marker of low metastatic potential in medullary cancer. These results are consistent with previous observations that demonstrated weaker TIMP-2 staining in neoplastic cells of invasive MTC (30). The observed inverse correlation between MMP-9 and TIMP-2 expression may indicate that these molecules are coregulated by a currently unknown mechanism.

Previous studies have shown that MTC exhibits moderate to strong staining of VEGF-A (32–34). In addition, the VEGF-A-mediated stimulation of VEGFR-2 autophosphorylation is crucial in mediating the effects of VEGF-A, including vasodilatation, endothelial cell migration and proliferation, and it has been considered as the key mediator of VEGF-induced angiogenesis (35). The current study extended previous studies on the role of VEGF-A and its receptors in MTC. A positive correlation was observed between VEGFR-2 staining, tumor size and TNM stages and an inverse correlation was identified between TIMP-2 expression and VEGFR-2 levels. This observation is particularly significant, since it appears to be a link between the TIMP pathways and the VEGF cascade in MTC. Previously, it has been shown, in other tumors, that endothelial cells may respond to angiogenic factors, such as VEGF, by decreasing the synthesis of TIMP-2 to facilitate tumor angiogenesis and metastasis (8,36).

The present study also compared the expression of angiogenic markers in sporadic and hereditary MTC groups. The marked TIMP-2 staining observed in the hereditary group may be due to an earlier diagnosis of the disease in these patients due to molecular screening in contrast to a more advanced tumor at the time of diagnosis in the sporadic group of patients. The inverse correlation between tumor size and stage also suggested that the presence of TIMP in the hereditary form of the disease is a marker of a less aggressive tumor.

In conclusion, the results of the current study suggest that MMPs are implicated in the development and maintenance of sporadic and hereditary MTC. Notably, TIMP-2 has been found to correlate with a less invasive MTC presentation and may be a marker of less aggressive disease. Consequently, these observations reinforce the potential advantage of compounds that inhibit the tumor angiogenic activity in MTC.

## Figures and Tables

**Table I:** Clinical characteristics of 77 patients with MTC.

Patient characteristics	Values
Age, years	35.6 (2.5–83.3)^a^
Females, n (%)	45 (58.4)
Tumor stage, n (%)	
I	17 (22.1)
II	24 (31.2)
III	22 (28.6)
IV	14 (18.2)
Hereditary/sporadic, n (%)	36 (46.8)/41 (53.2)
Calcitonin, pg/ml	262.0 (28.0–953.6)^a^
CEA, ng/ml	14.6 (2.4–52.6)^a^
Persistent disease, n (%)	38 (49.4)

a Median values (25–75th percentile).

MTC, medullary thyroid cancer; CEA, carcinoembryonic antigen.

**Table II:** Correlation between MMP-9 and TIMP-2 staining and TNM in patients with MTC.

	TNM stage (n)					
			
	I	II	III	IV	r_s_	P-value
MMP-9					−0.116	0.37
−	3	10	4	7		
+	7	11	7	4		
++	2	1	3	1		
Total	12	22	14	12		
TIMP-2					−0.395	0.001
−	2	1	3	3		
+	0	3	3	5		
++	2	4	3	2		
+++	8	16	7	1		
Total	12	24	15	12		

MMP-9, matrix metalloproteinase 9; TIMP-2 tissue inhibitor of metalloproteinase 2; TNM, tumor-node-metastasis; MTC, medullary thyroid cancer; r_s,_ Spearman’s rank correlation.

**Table III:** Correlation between MMP-9 and TIMP-2 staining with local or distant metastasis.

A, MMP-9							

Metastasis	n	−, n	+, n	++, n	+++, n	r_s_	P-value
Lymph node						0.005	0.96
N0	34	13	17	4	0		
N1	31	13	13	5	0		
Distant						−0.239	0.053
M0	52	17	27	8	0		
M1	14	9	4	1	0		

B, TIMP-2							

Metastasis	n	−, n	+, n	++, n	+++, n		P-value

Lymph node						0.423	0.0001
N0	14	2	3	6	24		
N1	24	9	9	6	9		
Distant						−0.416	0.001
M0	14	7	7	9	32		
M1	22	5	5	3	1		

MMP-9, matrix metalloproteinase 9; TIMP-2, tissue inhibitor of metalloproteinase 2; r_s,_ Spearman’s rank correlation; N, lymph node metastasis; M, distant metastasis.

**Table IV:** MMP-9 and TIMP-2 expression in hereditary and sporadic medullary thyroid carcinoma.

Variable	Hereditary, n	Sporadic, n	P-value
MMP-9			0.654
n	32	37	
−	14	14	
+	14	18	
++	4	5	
TIMP-2			0.001
n	34	38	
−	2	10	
+	3	9	
++	5	8	
+++	24	11	

Mann-Whitney U test. MMP-9, matrix metalloproteinase 9; TIMP-2, tissue inhibitor of metalloproteinase 2.

## References

[1] Roy R, Yang J, Moses MA (2000). Matrix metalloproteinases as novel biomarkers and potential therapeutic targets in human cancer. J Clin Oncol.

[2] Seo DW, Li H, Guedez L, Wingfield PT, Diaz T, Salloum R, Wei BY, Stetler-Stevenson WG (2003). TIMP-2 mediated inhibition of angiogenesis: an MMP-independent mechanism. Cell.

[3] Coussens LM, Fingleton B, Matrisian LM (2002). Matrix metalloproteinase inhibitors and cancer trials and tribulations. Science.

[4] Massi D, Franchi A, Ketabchi S, Paglierani M, Pimpinelli N, Santucci M (2003). Expression and prognostic significance of matrix metalloproteinases and their tissue inhibitors in primary neuroendocrine carcinoma of the skin. Hum Pathol.

[5] Bergers G, Brekken R, McMahon G (2000). Matrix metalloproteinase-9 triggers the angiogenic switch during carcinogenesis. Nat Cell Biol.

[6] Baker AH, Edwards DR, Murphy G (2002). Metalloproteinase inhibitors: biological actions and therapeutic opportunities. J Cell Sci.

[7] Rydlova M, Holubec L, Ludvikova M, Kalfert D, Franekova J, Povysil C, Ludvikova M (2008). Biological activity and clinical implications of the matrix metalloproteinases. Anticancer Res.

[8] Stetler-Stevenson WG, Seo DW (2005). TIMP-2: an endogenous inhibitor of angiogenesis. Trends Mol Med.

[9] Wang H, Wen Y, Mooney S, Li H, Behr B, Polan ML (2003). Matrix metalloproteinase and tissue inhibitor of matrix metalloproteinase expression in human preimplantation embryos. Fertil Steril.

[10] Dimo B, Ioannidis I, Karameris A, Vilaras G, Tzoumakari P, Nonni A, Patsouris E, Lazaris AC (2012). Comparative study of the Immunohistochemical expression of tissue inhibitors of metalloproteinases 1 and 2 between clearly invasive carcinomas and ‘in situ’ trophoblast invasion. Med Oncol.

[11] Imren S, Kohn DB, Shimada H, Blavier L, DeClerk YA (1996). Overexpression of tissue inhibitor of metalloproteinases-2 retroviral-mediated gene transfer in vivo inhibits tumor growth and invasion. Cancer Res.

[12] Bourboulia D, Jensen-Taubman S, Rittler MR, Han HY, Chatterjee T, Wei B, Stetler-Stevenson WG (2011). Endogenous angionesesis inhibitor blocks tumor growth via direct and indirect effects on tumor microenvironment. Am J Path.

[13] DeClerck YA, Perez N, Shimada H, Boone TC, Langley KE, Taylor SM (1992). Inhibition of invasion and metastasis in cells transfected with an inhibitor of mettaloproteinases. Cancer Res.

[14] Ceolin L, Siqueira DR, Romitti M, Ferreira CV, Maia AL (2012). Molecular basis of medullary thyroid carcinoma: the role of RET polymorphisms. Int J Mol Sci.

[15] Puñales MK, da Rocha AP, Meotti C, Gross JL, Maia AL (2008). Clinical and oncological features of children and young adults with multiple endocrine neoplasia type 2A. Thyroid.

[16] Kloos RT, Eng C, Evans DB, Francis GL, Gagel RF, Gharib H, Moley JF, Pacini F, Ringel MD, Schlumberger M, Wells SA (2009). Medullary thyroid cancer: management guidelines of the American Thyroid Association. Thyroid.

[17] Mulligan LM, Kwok JB, Healey CS, Elsdon MJ, Eng C, Gardner E, Love DR, Mole SE, Moore JK (1993). Germ-line mutations of the RET proto-oncogene in multiple endocrine neoplasia type 2A. Nature.

[18] Hundahl SA, Fleming ID, Fremgen AM, Menck HR (1998). A National Cancer Data Base report on 53,856 cases of thyroid carcinoma treated in the U.S., 1985–1995. Cancer.

[19] Mitchell JC, Parangi S (2005). Angiogenesis in benign and malignant thyroid disease. Thyroid.

[20] Maeta H, Ohgi S, Terada T (2001). Protein expression of matrix metalloproteinases 2 and 9 and tissue inhibitors of metalloproteinase 1 and 2 in papillary thyroid carcinomas. Virchows Arch.

[21] Siqueira DR, Romitti M, da Rocha AP, Ceolin L, Meotti C, Estivalet A, Puñales MK, Maia AL (2010). The RET polymorphic allele S836S is associated with early metastatic disease in patients with hereditary or sporadic medullary thyroid carcinoma. Endocr Relat Cancer.

[22] O’Sullivan B, Shah J (2003). New TNM staging criteria for head and neck tumors. Semin Surg Oncol.

[23] Nelson AR, Fingleton B, Rothenberg ML, Matrisian LM (2000). Matrix metalloproteinases: Biologic activity and clinical implications. J Clin Oncol.

[24] Kumamoto H, Yamauchi K, Yoshida M, Ooya K (2003). Immunohistochemical detection of matrix metalloproteinases (MMPs) and tissue inhibitors of metalloproteinases (TIMPs) in ameloblastomas. J Oral Pathol Med.

[25] Lee SJ, Tsang PS, Daz TM, Wei BY, Stetler-Stevenson WG (2010). TIMP-2 modulates VEGFR-2 phosphorylation and enhances phosphodiesterase activity in endothelial cells. Lab Invest.

[26] Cho Mar K, Eimoto T, Tateyama H, Arai Y, Fujiyoshi Y, Hamaguchi M (2006). Expression of matrix metalloproteinases in benign and malignant follicular thyroid lesions. Histopathology.

[27] Korem S, Kraiem Z, Shiloni E, Yehezkel O, Sadeh O, Resnick MB (2002). Increased expression of matrix metalloproteinase-2: a diagnostic marker but not prognostic marker of papillary thyroid carcinoma. Isr Med Assoc J.

[28] Albini A, Melchiori A, Santi L, Liotta LA, Brown PD, Stetler-Stevenson WG (1991). Tumor cell invasion inhibited by TIMP-2. J Natl Cancer Inst.

[29] Ring P, Johansson K, Höyhtyä M, Rubin K, Lindmark G (1997). Expression of tissue inhibitor of metalloproteinases TIMP-2 in human colorectal cancer - a predictor of tumour stage. Br J Cancer.

[30] Tomita T (1997). Matrix metalloproteinases and tissue inhibitors of metalloproteinases in thyroid C-cells and medullary thyroid carcinomas. Histopathology.

[31] Cavalheiro BG, Junqueira CR, Brandão LG (2008). Expression of matrix metalloproteinase 2 (MMP-2) and tissue inhibitor of metalloproteinase 2 (TIMP-2) in medullary thyroid carcinoma: prognostic implications. Thyroid.

[32] Capp C, Wajner SM, Siqueira DM, Brasil BA, Meurer L, Maia AL (2010). Increased expression of vascular endothelial growth factor and its rreceptors, VEGFR-1 and VEGFR-2 in medullary thyroid carcinoma. Thyroid.

[33] Bunone G, Vigneri P, Mariani L, Buto S, Collini P, Pilotti S, Pierotti MA, Bongarzone I (1999). Expression of angiogenesis stimulators and inhibitors in human thyroid tumors and correlation with clinical pathological features. Am J Pathol.

[34] de la Torre NG, Buley I, Wass JA, Turner HE (2006). Angiogenesis and lymphangiogenesis in thyroid proliferative lesions: relationship to type and tumour behaviour. Endocr Relat Cancer.

[35] Roy H, Bhardwaj S, Ylä-Herttuala S (2006). Biology of vascular endothelial growth factors. FEBS Lett.

[36] Lamoreaux WJ, Fitzgerald ME, Reiner A, Hasty KA, Charles ST (1998). Vascular endothelial growth factor increases release of gelatinase A and decreases release of tissue inhibitor of metalloproteinases by microvascular endothelial cells in vitro. Microvasc Res.

